# Thyroid diseases and bone health

**DOI:** 10.1007/s40618-017-0753-4

**Published:** 2017-08-29

**Authors:** G. R. Williams, J. H. D. Bassett

**Affiliations:** 1 Molecular Endocrinology Laboratory, Department of Medicine, Imperial College London, Hammersmith Campus, Du Cane Road, 10N5 Commonwealth Building, London, W12 0NN UK; 2 Molecular Endocrinology Laboratory, Department of Medicine, Imperial College London, Hammersmith Campus, Du Cane Road, 10N6 Commonwealth Building, London, W12 0NN UK

**Keywords:** Bone development, Osteoporosis, Hypothyroidism, Thyrotoxicosis, Thyroid hormone receptor α

## Abstract

Thyroid hormones are essential for skeletal development and are important regulators of bone maintenance in adults. Childhood hypothyroidism causes delayed skeletal development, retarded linear growth and impaired bone mineral accrual. Epiphyseal dysgenesis is evidenced by classic features of stippled epiphyses on X-ray. In severe cases, post-natal growth arrest results in a complex skeletal dysplasia. Thyroid hormone replacement stimulates catch-up growth and bone maturation, but recovery may be incomplete dependent on the duration and severity of hypothyroidism prior to treatment. A severe phenotype characteristic of hypothyroidism occurs in children with resistance to thyroid hormone due to mutations affecting *THRA* encoding thyroid hormone receptor α (TRα). Discovery of this rare condition recapitulated animal studies demonstrating that TRα mediates thyroid hormone action in the skeleton. In adults, thyrotoxicosis is well known to cause severe osteoporosis and fracture, but cases are rare because of prompt diagnosis and treatment. Recent data, however, indicate that subclinical hyperthyroidism is associated with low bone mineral density (BMD) and an increased risk of fracture. Population studies have also shown that variation in thyroid status within the reference range in post-menopausal women is associated with altered BMD and fracture risk. Thus, thyroid status at the upper end of the euthyroid reference range is associated with low BMD and increased risk of osteoporotic fragility fracture. Overall, extensive data demonstrate that euthyroid status is required for normal post-natal growth and bone mineral accrual, and is fundamental for maintenance of adult bone structure and strength.

## Bone cells

Chondrocytes, osteoblasts, osteocytes and osteoclasts comprise the four major cell types in the skeleton. Cartilage-forming chondrocytes, bone-forming osteoblasts and terminally differentiated osteocytes are derived from mesenchyme whereas bone-resorbing multinucleated osteoclasts differentiate from precursor cells of the monocyte/macrophage lineage. Chondrocytes and osteoblasts are directly responsive to thyroid hormone and, whilst osteoclast activity is also sensitive to changes in thyroid status, it remains uncertain whether osteoclasts are direct target cells or whether the effects of thyroid hormone on bone resorption are indirect and mediated via primary thyroid hormone actions in other cell types. The effects of thyroid hormone on osteocyte function are unknown [1].

## Bone formation and growth

**Fig. 1:**
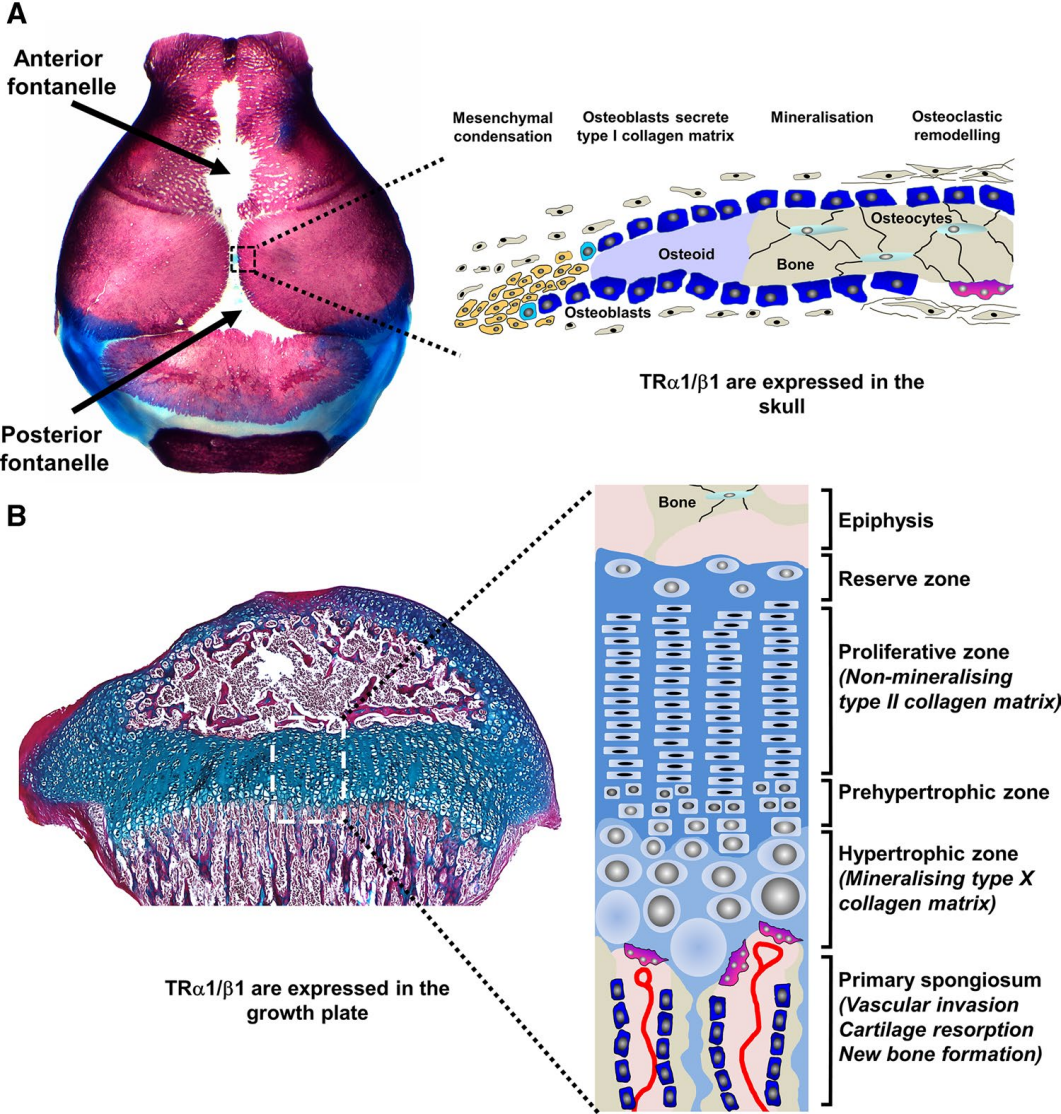
Intramembranous and endochondral ossification. **a** Skull vault stained with alizarin red (bone) and alcian blue (cartilage) from post-natal day 1 showing anterior and posterior fontanelles and sutures. Schematic representation of intramembranous bone formation at a skull suture. **b** Proximal tibial growth pate section at post-natal day 21 stained with alcian blue (cartilage) and van Gieson (bone matrix, *red*). Schematic representation of endochondral ossification showing chondrocyte proliferation, differentiation and apoptosis within the growth plate

During skeletal development, bone formation occurs via two distinct processes (Fig. 1). The flat bones of the skull vault and pelvis, together with the lateral third of the clavicle, form by intramembranous ossification in which mesenchyme progenitor cells condense and differentiate directly into osteoblasts [2]. Long bones and vertebrae, by contrast, form on an intermediary cartilage template by endochondral ossification after mesenchyme precursors differentiate to chondrocytes [3]. Epiphyseal growth plates form at the ends of developing long bones and contain chondrocytes that organize into distinct reserve, proliferative, pre-hypertrophic and hypertrophic zones (Fig. 1). Progression of reserve zone progenitor cells through the proliferative and hypertrophic zones is accompanied by a large increase in cell volume that is responsible for linear growth. Differentiating chondrocytes secrete a cartilage matrix that is rich in collagen types II and X. They ultimately undergo apoptosis and release growth factors and cytokines that stimulate vascular invasion and the migration of osteoblasts and osteoclasts, which model and mineralize the developing bone during growth [4]. Linear growth progresses until puberty when the growth plates fuse, but bone mineral accrual continues until peak bone mass is achieved during the third decade [5].

## Adult bone maintenance

**Fig. 2:**
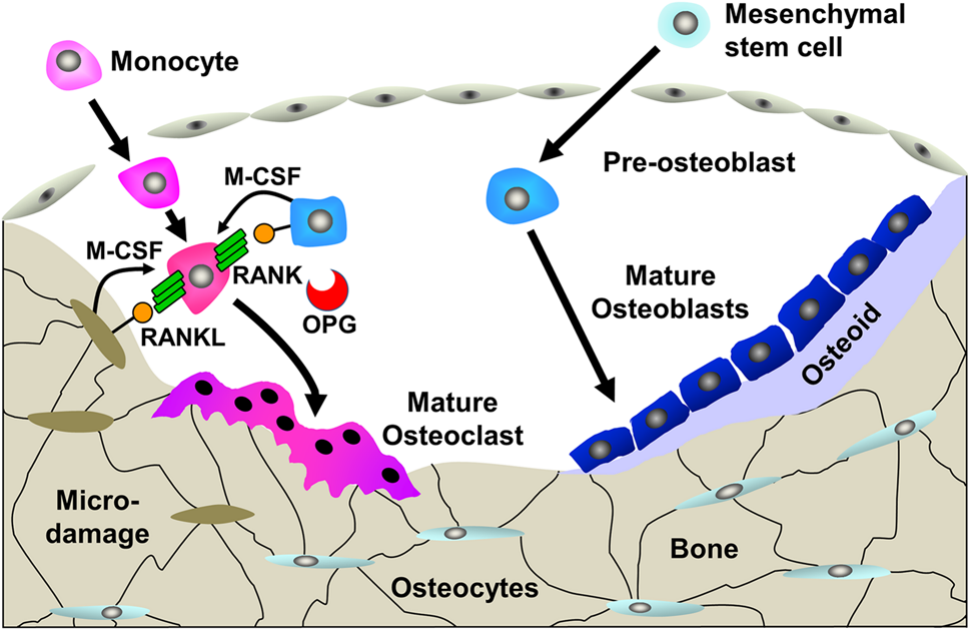
Bone remodelling cycle. The bone remodelling cycle is orchestrated by osteocytes that are entombed within the bone structure. Bone remodelling is initiated by changes in mechanical load, structural micro-damage or exposure to systemic or paracrine factors. Monocyte/macrophage precursors differentiate to mature osteoclasts and resorb bone. Differentiation is induced by macrophage colony-stimulating factor (M-CSF) and receptor activator of NFkB ligand (RANKL) and inhibited by osteoprotegerin (OPG). Following resorption, osteoblastic progenitors are recruited, synthesize an osteoid matrix and regulate its mineralization to form new bone and thus repair the defect

In adults, the structure and function of the skeleton are maintained by a process of continuous repair, mediated by the bone remodelling cycle (Fig. 2) [6]. Osteocytes are terminally differentiated cells of the osteoblast lineage that become entombed within mature bone. They sense mechanical strain and bone micro-damage, and orchestrate the activities of bone-resorbing osteoclasts and bone-forming osteoblasts within a coordinated cycle of bone resorption and formation [7]. Bone resorption and formation are coupled in time and space, and so targeted bone modelling and remodelling enables the adult skeleton to repair and replace old and damaged tissue in response to injury and mechanical loading, or react quickly to the demands of mineral homeostasis [8]. Uncoupling of these processes leads to accelerated bone loss in osteoporosis or accumulation of bone in osteopetrosis.

## Thyroid hormone action

**Fig. 3:**
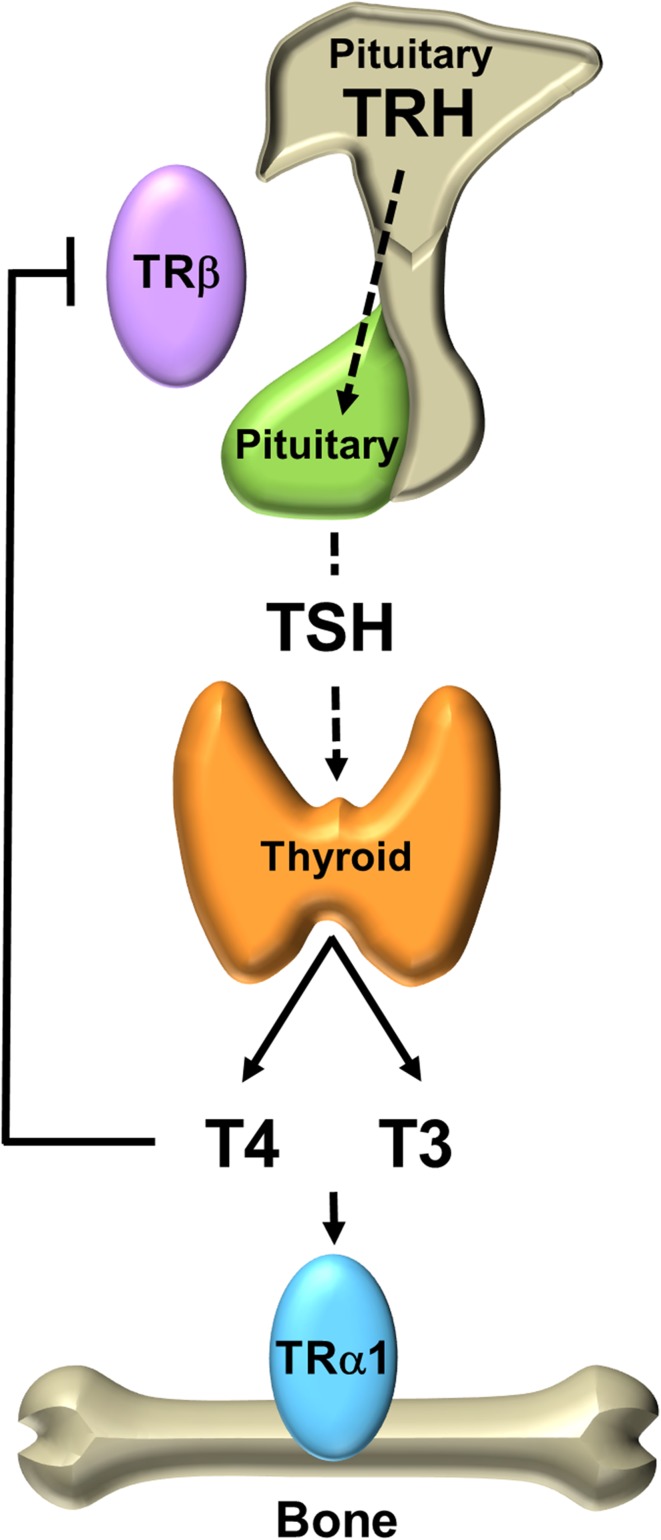
Hypothalamic–pituitary–thyroid axis. The thyroid gland secretes the pro-hormone T4 and the active hormone T3 and circulating concentrations are regulated by a classical endocrine negative feedback loop that maintains an inverse physiological relationship between TSH, and T4 and T3

Thyroid hormones have important effects on skeletal development, linear growth and the maintenance of adult bone mass and strength. The thyroid gland mainly secretes thyroxine (3,5,3′,5′-l-tetraiodothyronine, T4), and the circulating level of T4 is approximately fourfold higher than the concentration of the biologically active hormone 3,5,3′-l-triiodothyronine (T3) [9]. A classic endocrine negative feedback loop (Fig. 3) maintains an inverse relationship between the circulating concentrations of thyroid hormones and thyroid stimulating hormone (thyrotropin, TSH), thus establishing the hypothalamic–pituitary–thyroid (HPT) axis set-point [10, 11].

**Fig. 4:**
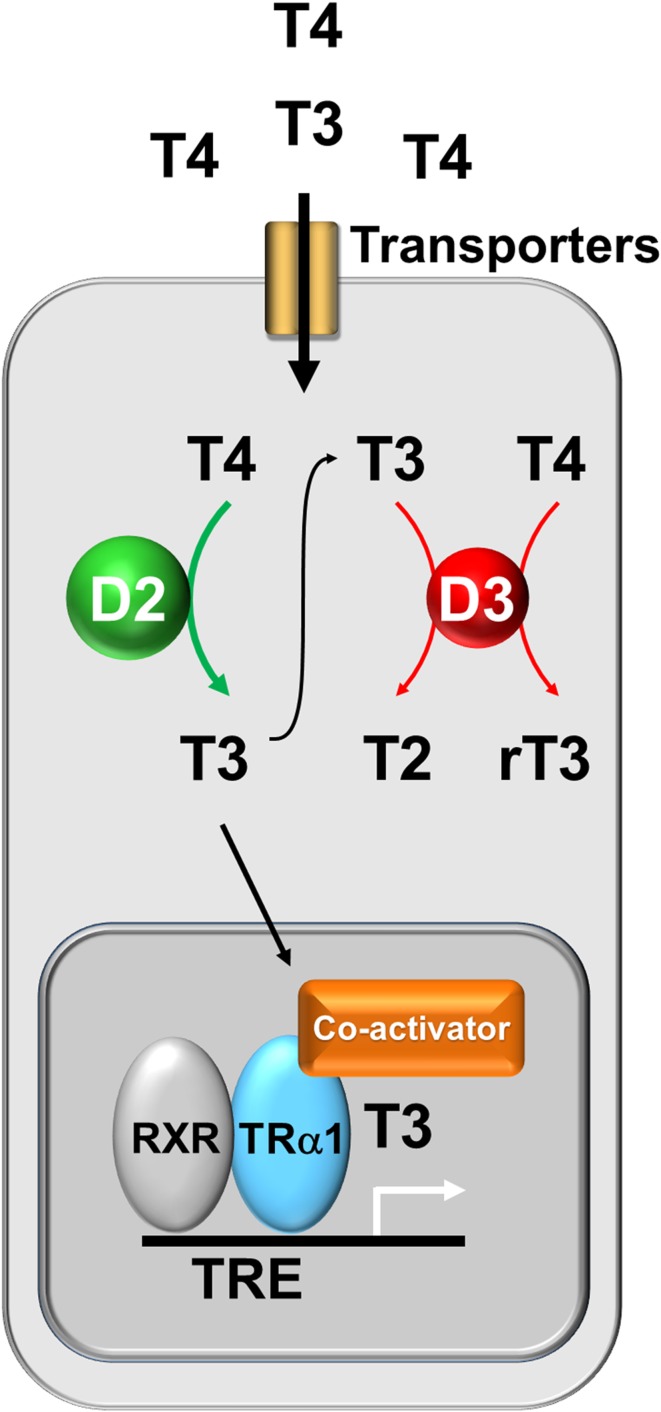
Thyroid hormone action in bone cells. Thyroid hormones enter T3 target cells via specific membrane transporters. The relative activities of the type 2 and type 3 deiodinases (D2 and D3) are regulated to ensure optimal intracellular T3 availability resulting in the displacement of the co-repressor and the binding of the co-activator and thus the physiological transcriptional activity of TRα1

T4 and T3 enter target cells via active transport involving several specific transporter proteins, including monocarboxylate transporters 8 and 10 (MCT8, MCT10), organic anion transporter protein 1c1 (OATP1c1), and the l-type amino acid transporters 1 and 2 (LAT1, LAT2) [12]. Once inside the target cell, T4 and T3 are metabolized by either the activating type 2 iodothyronine deiodinase (DIO2) or the inactivating type 3 enzyme (DIO3). DIO2 catalyses 5′-deiodination of T4 to generate the active hormone T3, whereas DIO3 catalyses removal of the 5-iodine atom from T4 or T3 to generate the inactive metabolites, reverse T3 (3,3′,5′-l-triiodothyronine, rT3) or 3,3′-diiodotyrosine (T2), respectively. Thus, the balance of DIO2 and DIO3 activities regulates the intracellular supply of the active hormone, T3 [9, 13]. T3 then enters the nucleus where it binds and activates either thyroid hormone receptor α or β (TRα, TRβ) (Fig. 4). TRs function as hormone-dependent transcription factors that repress target gene expression in the absence of hormone and stimulate gene transcription in response to T3 binding [14]. TRβ is the main receptor expressed in the hypothalamus and pituitary where it mediates negative feedback control of the HPT axis, whereas TRα is the main receptor expressed in the skeleton and mediates T3 action in bone and cartilage [1].

## Skeletal consequences of thyroid disease in children

### Hypothyroidism

Hypothyroidism in childhood is relatively common and causes delayed skeletal development, growth retardation and short stature with impaired bone maturation due to defective endochondral ossification. Impaired intramembranous ossification results in delayed closure of the fontanelles, persistently patent skull sutures and a typically flat nasal bridge and broad face. In severe undiagnosed cases, there is complete post-natal growth arrest and skeletal dysplasia with characteristic X-ray features that include stippled epiphyses reflecting epiphyseal dysgenesis, congenital hip dislocation, vertebral immaturity, scoliosis, patent fontanelles and sutures with delayed tooth eruption [1, 15].

Prompt treatment of children with thyroid hormone replacement induces a period of rapid “catch-up” growth in which skeletal maturation and bone age are also accelerated. Ultimately, normal adult height and bone mineral density (BMD) can be expected [16]. However, predicted adult height may not always be achieved and in such cases the deficit correlates with the severity of hypothyroidism and its duration prior to commencement of thyroid hormone replacement [17].

### Hyperthyroidism

Thyrotoxicosis in children is relatively rare and causes accelerated intramembranous and endochondral ossification and an increase in linear growth rate [1]. Paradoxically, the accompanying advancement in bone age may result in premature fusion of the growth plates and early cessation of growth leading to persistent short stature [18, 19]. In severe thyrotoxicosis in young children, early closure of the cranial sutures can result in craniosynostosis with neurological sequelae. Untreated hyperthyroidism during pregnancy is also associated with craniosynostosis and may be a causative risk factor [20].

### Summary of skeletal consequences of thyroid disease in children

Overall, T3 acts via TRα in chondrocytes and osteoblasts to regulate intramembranous and endochondral ossification and control the rate of linear growth and bone maturation and mineralization. Hypothyroidism in childhood causes delayed skeletal development and mineralization but prompt treatment with thyroid hormone initiates “catch-up” growth and stimulates bone maturation. Thyrotoxicosis, by contrast, accelerates skeletal development and bone mineral deposition, but may also result in short stature. Thus, the opposing skeletal consequences of childhood hypothyroidism and thyrotoxicosis, together with the rapid response of the hypothyroid skeleton to thyroid hormone replacement, demonstrate that normal euthyroid status during growth and adolescence is essential to establish peak bone mass and strength in early adulthood [1].

## Skeletal consequences of thyroid disease in adults

A large number of studies investigating the influence of thyroid disease on the adult skeleton have been published and reviewed. However, overall interpretation of findings from these studies is limited by heterogeneous study design, confounding factors, lack of power and differing end-point analyses [1]. Nevertheless, large studies including extensive individual participant meta-analyses have been performed and this review will concentrate on the larger and more recent studies that have strengthened our understanding.

### Hypothyroidism

Early histomorphometry analysis demonstrated that hypothyroidism results in low bone turnover with decreased osteoblastic bone formation and reduced osteoclastic bone resorption. The prolonged bone remodelling cycle includes a longer period of secondary mineralization resulting in a net increase in bone mineralization and mass without a change in bone volume [21, 22]. These changes are very slow, and long-term prospective follow-up of untreated hypothyroid patients would be required to demonstrate clinically significant changes in BMD by non-invasive imaging techniques. Thus, there are no good clinical data that demonstrate the skeletal consequences of hypothyroidism in adults.

### Hyperthyroidism

Hyperthyroidism, in contrast, causes high bone turnover with an increased frequency of initiation of bone remodelling sites together with increased bone resorption and formation rates. The remodelling time is shortened with imbalance between resorption and formation that results in a net loss of approximately 10% of bone per remodelling cycle [23, 24]. Thus, hyperthyroidism is an established cause of high bone turnover with accelerated bone loss leading to osteoporosis and increased fracture susceptibility. Severe osteoporosis resulting from untreated thyrotoxicosis is now rare because of early diagnosis and treatment, although undiagnosed hyperthyroidism is an important risk factor for secondary bone loss and osteoporosis in patients presenting to hospital with fracture [25, 26]. Accordingly, population studies have demonstrated an increased risk of fracture in thyrotoxicosis especially in post-menopausal women [27–31].

### Subclinical thyroid disease

Subclinical hypothyroidism is defined biochemically as the occurrence of circulating concentrations of T4 and T3 within their normal reference ranges in the presence of a TSH level elevated above its reference range. Conversely, subclinical hyperthyroidism occurs when T4 and T3 levels are within their reference ranges, but the TSH concentration is suppressed below its reference range. The effect of subclinical hypothyroidism on bone mineralization and fracture susceptibility has not been studied extensively [1], but a recent individual participant meta-analysis of data from 70,298 subjects from 13 prospective cohort studies consisting of 762,401 person-years follow-up demonstrated no association with fracture [28].

More studies have investigated the relationship between subclinical hyperthyroidism and BMD or fracture risk [1] and large population studies have suggested an increase in bone turnover, decrease in BMD and an increased risk of fracture, especially in post-menopausal women, although heterogeneity between studies prevented firm conclusions in several meta-analyses and reviews [32–38]. Nevertheless, the recent large individual participant meta-analysis of data from 70,298 subjects demonstrated that a TSH value below 0.01 mU/L was associated with a 2- and 3.5-fold increased risk of hip and spine fractures, respectively. Overall, subclinical hyperthyroidism was associated with bone loss and fracture, particularly in individuals with endogenous disease [28]. Furthermore, a register-based cohort of 222,138 subjects with a normal TSH level plus 9217 individuals with a low TSH were followed up for 7.5 years. This study demonstrated an exponential association between the duration of thyrotoxicosis and fracture. In euthyroid individuals, the risk of fracture increased with each standard deviation unit decrease in TSH (hazard ratio 1.45, *p* < 0.001 for hip fracture; HR 1.32, *p* < 0.001 for major osteoporotic fracture) [39].

### Variation within the euthyroid reference range

These findings suggest the possibility that thyroid status even across the normal reference range is a continuous variable related to BMD and strength. A number of studies have been published to address this issue, but data have been conflicting, largely because of heterogeneity especially with regard to the age and gender of cohorts and differences in study size [40–51]. Recently, an individual participant meta-analysis was undertaken that included data from 56,835 subjects (*n* = 2565 with hip fracture) from 12 prospective cohort studies consisting of 659,059 person-years follow-up. This study demonstrated that lower TSH and higher fT4 within the reference range were associated with 22–25% increased risk of hip fractures [52]. Taken together, these studies indicate that thyroid status at within the upper end of the euthyroid reference range is associated with low BMD and an increased risk of fracture [1].

### TSH suppression therapy for thyroid cancer

Thyroid cancer cells express TSH receptor, and TSH stimulates cell proliferation, iodine uptake and thyroglobulin secretion from tumour cells. This is also true in metastatic cells in about 65% of cases. There is, therefore, a good rationale for TSH suppression therapy using thyroxine. Treatment has been shown to reduce recurrence and disease-specific mortality in retrospective studies [53, 54], whilst a prospective non-randomized study demonstrated that a lesser degree of TSH suppression is an independent predictor of disease progression in patients at high-risk of recurrent disease [55].

In pre-menopausal women, three systematic literature reviews showed that suppressive treatment with T4 had no effect on BMD [32, 35, 36] and two meta-analyses demonstrated no effect on BMD at the hip, lumbar spine or radius [34, 37]. No fracture data are available.

In post-menopausal women, three systematic literature reviews demonstrated conflicting effects of suppressive treatment with T4 on BMD [32, 35, 36], while two meta-analyses showed BMD was reduced by 7% at the lumbar spine (CI 4–10%) and 5% at the femur (CI 2–8%) [34, 37]. No prospective fracture data are available, but hospital admission data for patients with fracture demonstrated that the incidence of admissions for fracture was 2.5× higher in patients with TSH <0.05 mU/l [29]. In patients with hyperthyroidism, there was a 3× increase in hip and 4× increase in vertebral fracture if TSH was suppressed <0.01 mU/l [27].

Overall, post-menopausal women on suppressive doses of T4 may be at risk of bone loss and osteoporosis [1].

### Summary of skeletal consequences of thyroid disease in adults

Thyroid hormones act via TRα in osteoblasts, but their actions in osteocytes and osteoclasts have not been defined [1]. Thyroid hormones stimulate adult bone turnover via increased osteoclastic bone resorption. In euthyroid subjects, variation across the reference ranges for thyroid hormones and TSH is associated with bone loss. Thus, thyroid status at the upper end of the normal reference range is associated with lower BMD and an increased risk of fragility fracture. Thyrotoxicosis is a well-established cause of high bone turnover osteoporosis, resulting in an increased susceptibility to fracture. This complication is now rare because of prompt diagnosis and treatment. Subclinical hyperthyroidism is also associated with an increased risk of fracture in both men and women, but especially those with endogenous disease. TSH suppression therapy in thyroid cancer may be associated with bone loss and fracture in post-menopausal women. There is potential for the use of bisphosphonates to prevent bone loss in post-menopausal women at risk of fracture who are receiving long-term TSH suppression therapy [56]. There are no prospective studies in this area but, although the adverse side-effect of atrial fibrillation in patients taking bisphosphonates is controversial, it could be important as exposure to excess thyroid hormones in the elderly is also associated with AF and cardiovascular mortality [57, 58]. Thus, despite preclinical studies supporting the potential use of bisphosphonates for prevention of bone loss in post-menopausal women at risk of fracture who are exposed to thyroid hormone excess [56], other anti-resorptive drugs may theoretically be used according to the latest treatment guidelines [59]. Nevertheless, targeted prospective clinical trials are still needed to provide a clear evidence base for therapeutic intervention.

Overall, and in contrast to the effects on the juvenile skeleton in which thyroid hormones are anabolic and stimulate bone growth and mineralization, T3 exerts catabolic actions in the adult skeleton and stimulates bone loss [1].

## Skeletal consequences of genetic disorders of thyroid function

### Mutations affecting TSH (TSHB) and TSH receptor (TSHR)

Loss-of-function mutations in *TSHB*, encoding the TSHβ subunit, result in biologically inactive TSH and congenital hypothyroidism (OMIM 275100). Loss- and gain-of-function mutations in *TSHR*, encoding the TSH receptor, result in TSH resistance with congenital hypothyroidism (OMIM 275200) or autosomal dominant hyperthyroidism (OMIM 609152), respectively. Few reports have documented the skeletal consequences of these genetic disorders because children are treated early and the long-term outcomes reflect the response to thyroid hormone supplementation or thyroidectomy/ablation [1]. Nevertheless, normal growth occurs in individuals with congenital hypothyroidism following proper thyroid hormone replacement [60–62], and improvement of skeletal developmental manifestations is seen in patients with autosomal dominant hyperthyroidism following thyroidectomy and normalization of thyroid status [63, 64]. Overall, the limited available studies demonstrate that persistent absence of TSH signalling during normal skeletal development following adequate thyroid hormone replacement does not affect bone mineralization in children, whilst constitutive activation of the TSHR is not detrimental for skeletal development and maturation in patients who have been treated and had their normal euthyroid status restored [1].

### Mutations affecting thyroid hormone receptors

#### Resistance to thyroid hormone α (RTHα) (OMIM 614450)

The key role of TRα in the human skeleton has been exemplified by discovery of a new syndrome of resistance to thyroid hormone due to dominant-negative mutations in *THRA* (RTHα) [65]. The skeletal manifestations of this condition (OMIM 614450) are consistent with the characteristic consequences of congenital and juvenile hypothyroidism, and reflect impaired T3 action in cartilage and bone. To date, 15 *THRA* mutations in 11 separate codons affecting 29 individuals from 16 families have been described. Nine result in the expression of a mutant TRα1 protein alone, and six affect both TRα1 and TRα2 [66–76]. Affected individuals have normal serum TSH with low/normal T4, high/normal T3 concentrations and characteristically elevated fT3:fT4 and T3:rT3 ratios [77, 78].

Children with RTHα due to expression of mutant TRα1 alone have skeletal dysplasia manifest by variable features that include delayed bone age and tooth eruption, patent skull sutures with wormian bones and delayed closure of the fontanelles, macrocephaly, flattened nasal bridge, hypertelorism, disproportionate (sub-ischial) short stature mainly affecting the lower limbs, epiphyseal dysgenesis, acetabular hypoplasia, congenital hip dislocation, vertebral ossification defects and defective bone mineralization [66, 67, 71, 73]. Adults have been studied less comprehensively, but variable skeletal abnormalities include increased cortical bone mass, disproportionate short stature, macrocephaly with skull vault thickening and hearing loss due to otosclerosis [67, 70, 73, 74]. As the number of reported cases has increased, a phenotype–genotype correlation has emerged. Missense mutations that impair T3 binding and result in only mild or moderate dominant-negative activity of mutant TRα1 are associated with fewer and less severe abnormalities. By contrast, nonsense mutations resulting in the expression of a truncated TRα1 protein with absent T3 binding and potent dominant-negative activity result in marked skeletal dysplasia and developmental delay. Accordingly, T4 treatment of children harbouring severe mutations has had little effect on growth and skeletal development, whereas treatment of individuals with milder missense mutations has been more promising [66, 67, 70, 71, 73, 74, 76].

More recently, *THRA* mutations affecting both TRα1 and TRα2 have been described [67, 69, 72, 76]. Affected children and adults had a similar spectrum of abnormalities characteristic of the skeletal dysplasia and variable response to T4 treatment reported in individuals with mutations affecting TRα1 alone. In vitro analysis of the functional defects of mutant TRα1 proteins was also consistent with the phenotype–genotype correlation described above, while all studies showed that wild-type and mutant TRα2 proteins were transcriptionally inactive and had no dominant-negative activity [67, 69, 72, 76]. Nevertheless, a severe and atypical phenotype was described in a 27-year-old woman with an N359Y substitution affecting both TRα1 and TRα2 [68]. Her unique combination of skeletal abnormalities includes intrauterine growth retardation and failure to thrive, macrocephaly, hypertelorism, micrognathia, short and broad nose, agenesis of the clavicles and 12th ribs, elongated thorax, ovoid vertebrae, scoliosis, congenital hip dislocation, short limbs and dwarfism, unilateral humero-radial synostosis, elbow dislocation and syndactyly. The TRα1^N359Y^ mutant protein had impaired T3 binding and transactivation function with moderate dominant-negative activity, while no clear functional abnormality of the TRα2^N359Y^ mutant was identified [68, 79].

Together, these reports define a new genetic disorder, RTHα, characterized by profound and consistent developmental abnormalities of the skeleton that recapitulate findings in mice with dominant-negative *Thra* mutations [80–85]. Overall, similar phenotypes in patients with mutations affecting either TRα1 alone, or both TRα1 and TRα2, suggest TRα2 has little or no physiological role in the skeleton. However, the unique phenotype in the patient with the N359Y substitution affecting both TRα1 and TRα2, in whom no other de novo mutations could be identified by whole exome sequencing [68], raises the intriguing possibility that TRα2 may fulfil an unrecognized function during skeletal development.

#### Resistance to thyroid hormone β (RTHβ) (OMIM 188570)

RTHβ results from dominant-negative mutations of *THRB* that cause disruption of the HPT axis leading to a characteristic increase in TSH along with inappropriately normal or increased circulating T4 and T3 levels [86–88]. The clinical syndrome is complex with a mixed phenotype comprising hyperthyroid-like responses in some tissues and hypothyroid effects in others. These variable tissue responses depend on several factors including genetic background, the severity of the *THRB* mutation, the relative amounts of expressed mutant and wild-type TRβ proteins and tissue-specific differences in the ratio of TRα and TRβ protein expression. In addition to these confounding issues, patients may have received varying prior management including treatment with anti-thyroid drugs, thyroid hormone analogues or surgery. In this context, it is not surprising that descriptions of skeletal abnormalities in RTHβ are variable and largely restricted to case reports or case series. Long-term prospective studies in large families will be necessary to advance understanding [1]. Nevertheless, analysis of the skeletal consequences of mutations of *Thrb* in mouse models of RTHβ is consistent with a skeletal phenotype of accelerated bone development in juveniles and increased bone turnover with osteoporosis in adults that is due to increased T3 action in cartilage and bone [1, 83, 89–91].
